# Huge Trombus including Left Renal Vein, Ovarian Vein, and Inferior Vena Cava Mimicking Renal Colic

**DOI:** 10.1155/2014/351270

**Published:** 2014-07-22

**Authors:** Sakir Ongun, Sermin Coban, Abdullah Katgi, Funda Obuz, Aykut Kefi

**Affiliations:** ^1^Dokuz Eylul University School of Medicine, Department of Urology, 35340 Izmir, Turkey; ^2^Dokuz Eylul University School of Medicine, Department of Nephrology, 35340 Izmir, Turkey; ^3^Dokuz Eylul University School of Medicine, Department of Haematology, 35340 Izmir, Turkey; ^4^Dokuz Eylul University School of Medicine, Department of Radiology, 35340 Izmir, Turkey

## Abstract

A 31-year-old female presented with acute left flank pain; she had a C/S at the postpartum day 24. Ureteral stone was suspected but ultrasound examination was normal. Then Doppler ultrasound revealed a trombus in left renal vein and inferior vena cava. Contrast enhanced MDCT scan showed swelled and nonfunctional left kidney, a trombus including distal part of left ovarian vein, left renal vein, and inferior vena cava. We started anticoagulation treatment. Further examination revealed diagnosis of chronic myeloproliferative disease. The trombus was completely recanalized at 3-month followup.

## 1. Introduction

Ovarian vein thrombosis (OVT) is a rare and serious situation that affects mostly postpartum women, with a reported incidence of 0.02–0.20% of all pregnancies [1].

OVT occurs 80%–90% in the right side; this could be caused by compression of the right ovarian vein against the sacral promontory due to an enlarged dextroverted uterus and presence of retrograde flow in the left ovarian vein [2, 3].

Several risk factors have been identified: puerperium, multiparity, postoperative period, and underlying diseases like Crohn's disease, malignant tumor, systemic lupus proteins C and S, thrombophilia, and hyperhomocysteinemia [4].

The majority of cases remain misdiagnosed because of their nonspecific clinical presentation which can mimic pyelonephritis, ureteral obstruction, and acute abdomen [1, 5]. A high index of suspicion is required for diagnosis. We present an interesting case of a postpartum woman, who had a thrombus including distal part of left ovarian vein, left renal vein, and inferior vena cava.

## 2. Case Presentation

**Figure 1:**
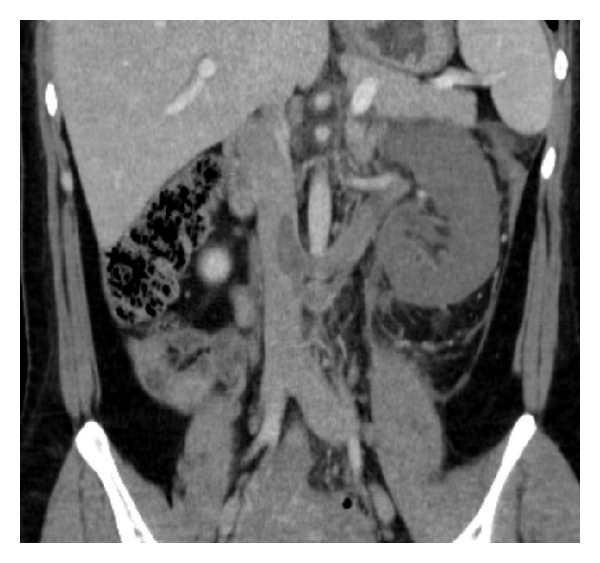
Swelled and nonfunctional left kidney and a thrombus including left renal vein and inferior vena cava.

**Figure 2:**
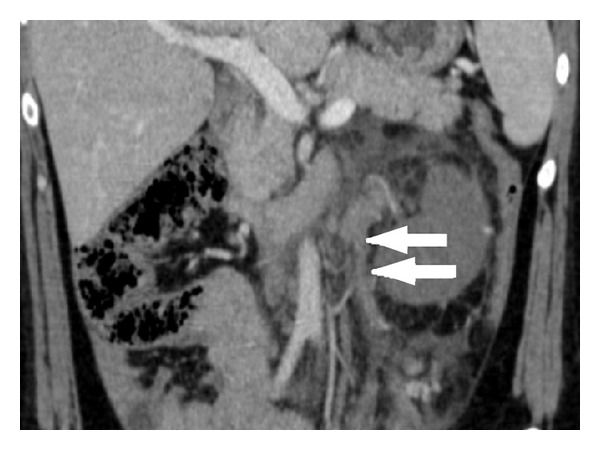
A thrombus including distal part of left ovarian vein (arrows).

A 31-year-old female presented with acute left flank pain; she was admitted to external center and treated as having ureteral stone. But her pains get worse in four days and she was admitted to our hospital. She had no fever but vomiting. She was at the postpartum day 24; she had a C/S and a live born-term male. In her clinical history, she had HELLP syndrome before pregnancy five years ago and 29 weeks of still birth. Blood pressure was 140/75 mmHg, heart rate was 70/min, and temperature was 36,5°C. White cell count was 9800/mm^3^ with 84% neutrophils. Urinalysis was normal. Ultrasound examination was normal but Doppler ultrasound revealed a thrombus in left renal vein and inferior vena cava. Contrast enhanced MDCT scan showed swelled and nonfunctional left kidney, a thrombus including distal part of left ovarian vein, left renal vein, and inferior vena cava (Figures 1 and 2).

The patient was hospitalized and intravenous heparin was commenced. Fibrinolytic therapy or immediate surgery was not planned because the episode started four days ago. After treatment with heparin, oral warfarin was started. Further examination revealed jak2 v617f mutation then bone marrow biopsy diagnosed chronic myeloproliferative disease and discharged with oral warfarin. The thrombus was completely recanalized at 3-month followup.

## 3. Discussion

OVT is an uncommon complication in the early postpartum, cesarean delivery and also increases the risk of thrombosis to 1-2% and multiparity has been identified as a risk factor for thrombosis in general [6, 7]. Although infrequent, OVT may progress to involve renal vein and inferior vena cava and may cause sepsis and pulmonary embolism all of which are potentially life threatening because of its nonspecific presentation [1, 6].

OVT may present with a broad range of symptoms, ranging from unresponsive fever to nonspecific back pain or right lower quadrant pain or may be entirely asymptomatic.

The most common presenting symptoms are fever (80%), pelvic pain (66%), and palpable abdominal mass (46%), often described as ropelike [1]. OVT can be secondary to one or more constituents of Virchow's triad, which include blood flow stasis, alteration in coagulation factors, and injury to the intima of blood vessels [1]. The most important risk factor is increased blood coagulation, which may be due to many predisposing factors, such as malignancies, puerperal fever, recent surgery, and immobilization [6]. Hypercoagulation conditions as systemic lupus erythematosus, antiphospholipid syndrome, presence of factor V Leiden, paroxysmal nocturnal haemoglobinuria, hyperhomocysteinemia, protein C and S deficiency, and heparin induced thrombocytopenia are all reported as risk factors for OVT [6, 8].

Complications of OVT are rare [8, 9] and can involve renal vein and VCI and lead to pulmonary embolism which is a life-threatening condition and has been reported in 13.2% of patients with OVT [1]. Ultrasound findings of OVT include an anechoic to hypoechoic mass between the adnexa and the inferior vena cava and the absence of blood flow within the mass. Ultrasonography can be used as a screening tool but should not be used as a primary study to rule out OVT without the aid of CT or MRI due to low sensitivity [5, 10]. Diagnostic imaging can be performed using ultrasound, CT scan, or MRI examinations, with magnetic resonance angiography having the best sensitivity and specificity [10].

The current clinical practice is to manage OVT conservatively. This includes antibiotics and anticoagulation with heparin [11]. Interestingly, our patient was afebrile at initial presentation and remained so during her hospitalization. She was therefore treated with anticoagulation only, because antibiotics were unnecessary.

In 80–90% of the cases the right ovarian vein is the one affected due to the incompetence of the valves [7]. To our knowledge this is the first case in the literature of left OVT including both RV and VCI, and the other reported few cases occurred on the right side [5, 12, 13].
